# Zearalenone Does Not Show Genotoxic Effects in the *Drosophila melanogaster* Wing Spot Test, but It Induces Oxidative Imbalance, Development, and Fecundity Alterations

**DOI:** 10.3390/toxins15060358

**Published:** 2023-05-25

**Authors:** Luis Felipe Santos-Cruz, Alberto Ponciano-Gómez, Juan Tomás Torres-Gregorio, Bertha Guadalupe Ramírez-Cruz, Gerardo Vázquez-Gómez, Luis Barbo Hernández-Portilla, Cesar Mateo Flores-Ortiz, Irma Elena Dueñas-García, María Eugenia Heres-Pulido, Laura Castañeda-Partida, Ángel Durán-Díaz, Myriam Campos-Aguilar, Santiago Cristobal Sigrist-Flores, Elías Piedra-Ibarra

**Affiliations:** 1 Toxicología Genética, Biología, Facultad de Estudios Superiores Iztacala, Universidad Nacional Autónoma de México, Los Barrios N° 1, Los Reyes Iztacala, Tlalnepantla C.P. 54090, Mexico; 2 Laboratorio de Inmunología (UMF), Facultad de Estudios Superiores Iztacala, Universidad Nacional Autónoma de México, Los Barrios N° 1, Los Reyes Iztacala, Tlalnepantla C.P. 54090, Mexico; 3 Fisiología Vegetal (UBIPRO), Facultad de Estudios Superiores Iztacala, Universidad Nacional Autónoma de México, Los Barrios N° 1, Los Reyes Iztacala, Tlalnepantla C.P. 54090, Mexico; 4 Department of Cytokinetics, Institute of Biophysics of the Czech Academy of Sciences, 61265 Brno, Czech Republic; 5 Mathematics, Facultad de Estudios Superiores Iztacala, Universidad Nacional Autónoma de México, Los Barrios N° 1, Los Reyes Iztacala, Tlalnepantla C.P. 54090, Mexico

**Keywords:** zearalenone, *Drosophila melanogaster*, oxidative stress, cytochromes P450, wing spot test

## Abstract

Zearalenone (ZEN) is a non-steroidal mycoestrogen produced by the *Fusarium* genus. ZEN and its metabolites compete with 17-beta estradiol for cytosolic estrogen receptors, causing reproductive alterations in vertebrates. ZEN has also been associated with toxic and genotoxic effects, as well as an increased risk for endometrial adenocarcinomas or hyperplasia, breast cancer, and oxidative damage, although the underlying mechanisms remain unclear. Previous studies have monitored cellular processes through levels of transcripts associated with Phase I Xenobiotic Metabolism (*Cyp6g1* and *Cyp6a2*), oxidative stress (*hsp60* and *hsp70*), apoptosis (*hid*, *grim*, and *reaper*), and DNA damage genes (*Dmp53*). In this study, we evaluated the survival and genotoxicity of ZEN, as well as its effects on emergence rate and fecundity in *Drosophila melanogaster*. Additionally, we determined levels of reactive oxygen species (ROS) using the *D. melanogaster* flare and Oregon R(R)-flare strains, which differ in levels of *Cyp450* gene expression. Our results showed that ZEN toxicity did not increase mortality by more than 30%. We tested three ZEN concentrations (100, 200, and 400 μM) and found that none of the concentrations were genotoxic but were cytotoxic. Taking into account that it has previously been demonstrated that ZEN administration increased hsp60 expression levels and apoptosis gene transcripts in both strains, the data agree with an increase in ROS and development and fecundity alterations. Since *Drosophila* lacks homologous genes for mammalian estrogen receptors alpha and beta, the effects of this mycotoxin can be explained by a mechanism different from estrogenic activity.

## 1. Introduction

Zearalenone (ZEN), a mycotoxin produced by several Fusarium species [1], is a globally prevalent contaminant in many stored cereals, including corn, barley, oats, wheat, rice, and sorghum [1,2]. Regulatory limits on ZEN levels are set for various food products, ranging from 20 μg/kg to 100 μg/kg [3]. Despite a 1989 European Union ban, zearanol, a derivative of ZEN, continues to be used as a non-steroidal anabolic agent in some regions, such as in Mexico [4].

Orally administered ZEN is absorbed in the gut and transformed into α-zearalenol and β-zearalenol (α-ZOL, β-ZOL, respectively) metabolites with higher estrogenic activity than ZEN [5]. These metabolites can reach vital organs, acting as significant endocrine disruptors and affecting reproductive functions in vertebrates [6,7]. ZEN has also been linked to early puberty and certain cancers in humans [8].

Cytochrome P450 enzymes (Cyp450s) play a key role in the oxidation of various chemicals and substances, including ZEN [9]. ZEN can induce the expression of *Cyp3A4* in mammalian cells and can be metabolized via a process called monohydroxylation, possibly involving Cyp1A2 [10]. This process leads to metabolites, such as estrone and 17β-estradiol, that can cause genotoxic effects, such as oxidative DNA damage, which has been associated with an increased risk of cancer [11]. ZEN has demonstrated toxicity and genotoxic effects in various models. Studies have shown it can induce necrosis in human blood cells, reduce cell viability, and cause DNA damage in kidney epithelial cells [12,13]. It can also disrupt cell cycles and induce chromosomal aberrations. In mouse bone marrow cells and the HeLa cell lines ZEN and α-ZOL show greater genotoxicity than β-ZOL [14]. This evidence suggests that ZEN’s toxic metabolites remain harmful even after biotransformation.

The exact molecular mechanisms of ZEN’s toxicity and genotoxicity are still unclear. Current research is exploring this by examining gene expression changes in different organisms. ZEN can increase the activity of estrogen-dependent genes, Cyp450 enzymes, and heat shock proteins, and it can trigger cell death [15,16,17]. This indicates that the effects of ZEN and its metabolism are primarily mediated by its estrogenic activity. However, similar effects have been observed in animal models, such as *Drosophila melanogaster* [18]. In such insects, hormonal functions do not rely on estrogen, but rather on ecdysone in males and 20-hydroxydisone (20E) in females [19]. In the absence of an estrogen receptor, Drosophila melanogaster represents a model to study ZEA-induced non-estrogenic effects.

This study aimed to investigate the effects of ZEN on the survival of third instar larvae of the flare and Oregon R(R)-flare strains, having elevated concentrations of Cyp450s, which are involved in the hydroxylation of ZEN in various species, including humans. The study also aimed to assess the genotoxicity of ZEN at concentrations of 100, 200, and 400 μM using the *Drosophila melanogaster* wing spot test. In addition, the effects of ZEN on emergence rate, fecundity parameters, and levels of ROS were investigated at a concentration of 260 μM. The study also examined the expression of genes, such as *Cyp6g1*, *Cyp6a2*, *hsp60*, *hsp70*, *hid*, *grim*, *reaper*, and *Dmp53*, to evaluate the toxicity of ZEN and its impact on development and fertility.

## 2. Results

### 2.1. Survival Assays

**Figure 1:**
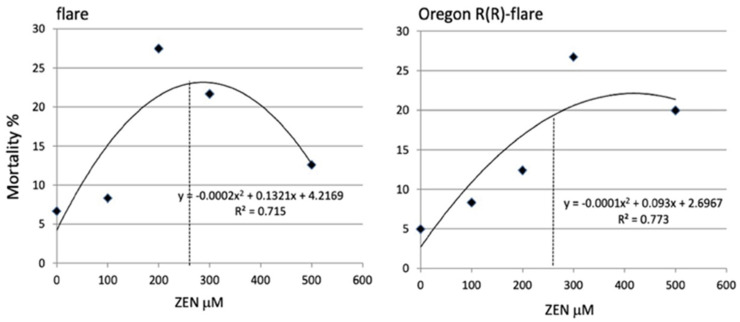
Mortality percentages of larvae fed with ZEN. Flare and Oregon R(R)-Flare third instar larvae were fed until pupation with medium supplemented with ZEN [0, 100, 200, 300, and 500 μM]. The dotted line indicates the mortality rate corresponding to 260 μM. The data were obtained from four independent experiments (with three replicates per treatment) and were analyzed using a two −way ANOVA with a significance level of *p* < 0.05.

An overall increase in mean mortality was observed in both the flare and Oregon R(R)-flare strains treated with ZEN [100, 200, 300, and 500 μM], in comparison to the PBS control. The mortality rate was lower in the Oregon R(R)-flare strain than in the flare strain. However, from 300 to 500 μM, the mortality rate decreased in a polynomial correlation. Therefore, we decided to test ZEN at 260 μM, a sublethal concentration that corresponded to a mortality rate between 20–25% in both strains, for the emergence and fecundity assays and gene expression profiling (Figure 1).

### 2.2. Genotoxicity Test

As expected, the pro-mutagen Urethane (URE, bioactivation and positive control) resulted in higher spot frequencies in the HB cross compared to the ST cross, due to the former’s high and inducible *Cyp450*s [20]. However, none of the concentrations of ZEN tested showed genotoxic effects, such as mutation, recombination, deletion, and aneuploidy [21], in both crosses (Table 1). Although we did not observe genotoxicity with this mycotoxin, statistical differences (*p* < 0.05) were found between the frequencies of small and total spots at 100 and 400 µM and between the small spot frequency in the 100 and 200 µM treatments in the ST cross with inducible *Cyp450*s. Notably, these frequencies decreased as the concentration increased (Table 1). Additionally, although we did not observe ZEN genotoxicity, the analysis of the accumulated mwh clone size class distribution of both crosses, using the Kolgomorov–Smirnov test (*p* < 0.05) [22], showed statistically significant differences between all treatments and the corresponding PBS dissolvent control, with a concentration-dependent decrease in the accumulated mwh clone size class distribution, but only in the ST cross (Table 2).

### 2.3. ZEN Identification and Quantification by HPLC in the Flare and Oregon R(R)-Flare Strains

Based on the HPLC analysis of three extract replicates from the flare and Oregon R(R)-flare imagos, a peak corresponding to the ZEN standard was detected, indicating that ZEN was incorporated into the larvae. The area under the curve was measured to quantify the amount of ZEN incorporated, and no statistical differences were found between the two strains. The mean concentration of ZEN in the flare strain was 3.77 mg/kg, while it was 5.86 mg/kg for the Oregon R(R)-flare strain, with standard deviations of +/− 2.0 and +/− 2.3, respectively (according to Supplementary Figure S1).

### 2.4. Emergence

**Figure 2:**
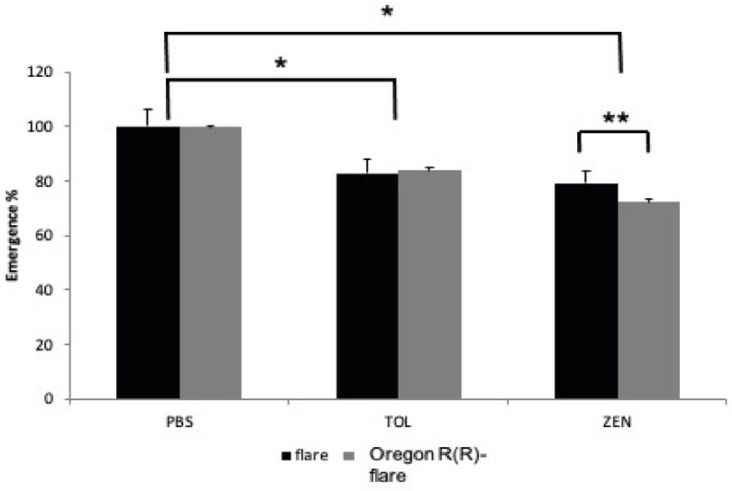
Emergence percentages of flare and Oregon R(R)-flare larvae. The larvae were fed until pupation with DIM supplemented with ZEN (260 μM) and Toluene (TOL) 50 mM dissolved in PBS pH 7. The asterisks over the lines indicate statistically significant differences. The data were analyzed using a two-way ANOVA with a significance level of *p* < 0.05 *, *p* < 0.01 **.

The percentage of emergence for each strain and treatment (Figure 2) showed statistically significant differences for the following comparisons: strains vs. ZEN (260 μM), PBS (dissolvent control) vs. toluene (TOL) 50 mM (positive control) and PBS vs. ZEN. In contrast, no significant differences in the emergence were observed between TOL and ZEN. The emergence of imagos from the flare strain treated with ZEN decreased 20.8% with respect to the PBS dissolvent control, while, in the Oregon R(R)-flare strain, the emergence decreased 27.7% with respect to the control. On the other hand, the emergence of imagos from the flare and Oregon R(R)-flare larvae treated with TOL decreased to 17% and 16% only, respectively.

### 2.5. Fecundity

**Figure 3:**
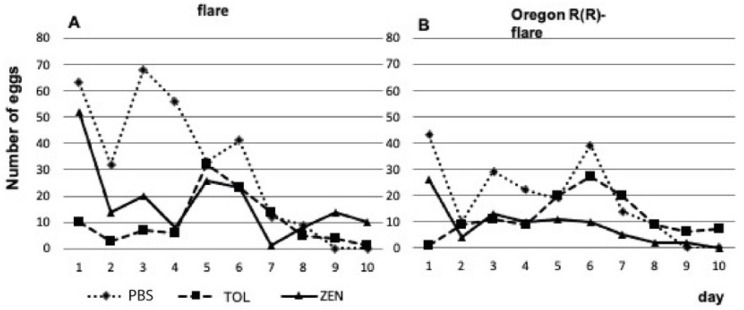
Fecundity of zearalenone-fed larvae. Daily egg laying by (**A**) flare and (**B**) Oregon R(R)-flare female flies fed with DIM supplemented with the dissolvent control PBS pH 7, TOL (50 mM) and ZEN (260 μM) dissolved in PBS pH 7.

**Figure 4:**
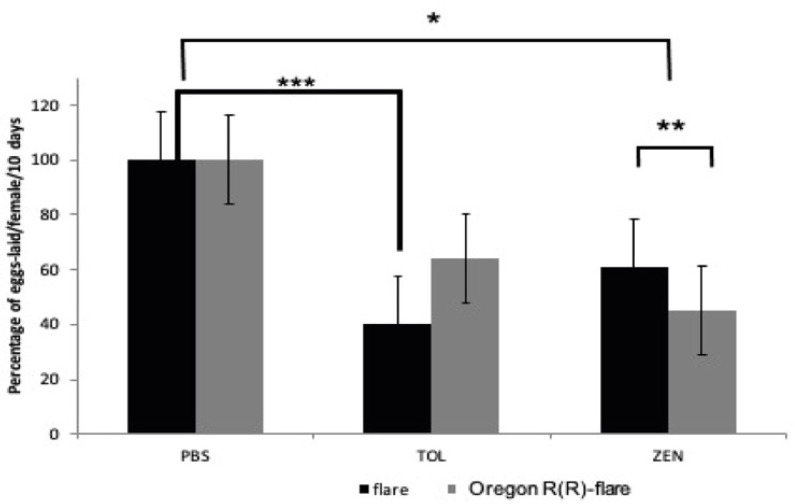
Percentages of eggs laid 0ver 10 days by flare and Oregon R(R)-flare female flies. The flies were fed with DIM supplemented with the dissolvent control PBS pH 7, Toluene 50 mM (TOL) and ZEN 260 μM dissolved in PBS. The asterisks above the lines indicate statistically significant differences. The data were analyzed using a two-way ANOVA with a significance level of *p* < 0.05 *, 0.01 **, 0.001 ***.

The mean daily egg-laying rate of female flies was compared between PBS dissolvent control, TOL (50 mM), and ZEN (260 µM) dissolved in PBS. Both flare (Figure 3A) and Oregon R(R)-flare (Figure 3B) strains treated with TOL and ZEN showed an overall decreasing trend in mean daily egg-laying rates compared to the PBS control, with the ZEN treatment resulting in a lower rate over 10 days. The total number of eggs laid by female flies from each strain was also recorded and the mean percentage calculated (Figure 4). Significant differences were observed between treatments and strains compared to the PBS control. The flare strain treated with TOL showed only 40% fecundity, while the Oregon R(R)-flare strain showed 64% fecundity. In the ZEN treatment, the flare strain had a higher fecundity (61%) than the Oregon R(R)-flare strain (45%).

### 2.6. Transcriptional Expression in Basal State

**Figure 5:**
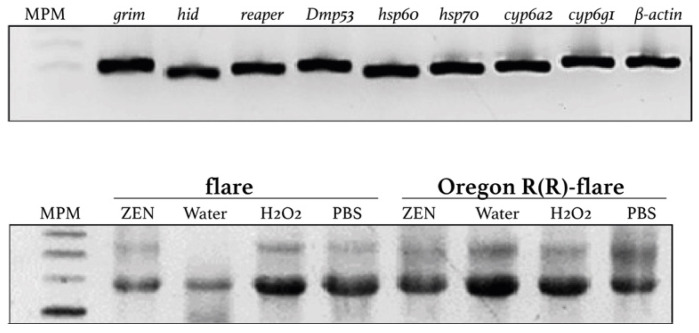
RT-PCR analysis of target genes. Flare and Oregon R(R)-flare larvae exposed to ZEN (260 µM) in dissolvent control (PBS pH 7), negative MilliQ water and H_2_O_2_ positive controls (lower panel). The upper panel shows the amplicons obtained using cDNA from the negative control and specific primers designed to amplify the xenobiotic metabolism, oxidative stress, apoptosis, and DNA damage biomarkers.

**Figure 6:**
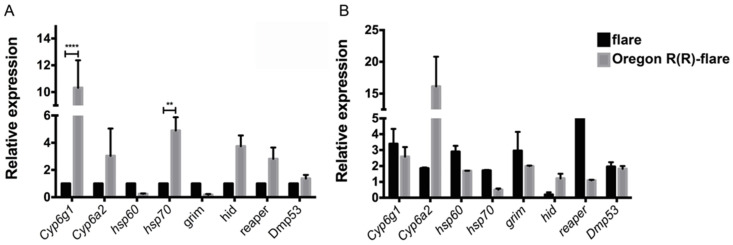
Relative expression levels of target genes. The relative expression levels of *Cyp6g1*, *Cyp6a2*, *hsp60*, *hsp70*, *hid*, *grim*, *reaper*, and *Dmp53* in third instar larvae of *D. melanogaster* flare and Oregon R(R)-flare strains under control conditions ((**A**), Reference sample was the flare strain) and treatment with ZEN (260 μM) dissolved in PBS pH 7 are shown ((**B**), Reference sample was the corresponding PBS-treated strain) (** *p* ≤ 0.01, **** *p* ≤ 0.0001, two-sided *t*-test).

Prior to evaluating the expression of transcripts, we verified the size of the amplifications for each oligonucleotide pair (Figure 5). Transcriptional expression of genes associated with XM, oxidative stress and apoptosis in the basal state revealed differences between the studied untreated strains. Our data indicated that the relative expressions of *Cyp6g1* and *Cyp6a2* genes were 10 and three times higher, respectively, in the larvae of the Oregon R(R)-flare strain compared to the flare strain (see Figure 6). Additionally, we observed contrasting expression levels of *hsp60* and *hsp70* genes, as the anti- and pro-apoptotic *hsp60* levels decreased in the Oregon R(R)-flare strain, while the pro-apoptotic *hsp70* levels increased approximately five times more than those in the flare strain. Similarly, the apoptosis genes transcripts showed contrasting expressions in the Oregon R(R)-flare strain, where the transcript abundance of *grim* was lower than in the flare strain, but the levels of hid and reaper were 3.74 and 2.81-fold higher than those in the flare strain exposed to similar conditions (see Figure 6A).

### 2.7. Transcriptional Expression Associated with ZEN Exposure

The transcript levels of genes related to XM, oxidative stress, and apoptosis were analyzed in untreated and ZEN-treated larvae from the flare and Oregon R(R)-flare strains. Figure 6B presents the gene expression data for the flare strain following ZEN administration, compared to control conditions (PBS pH7). The ZEN treatment in flare larvae led to increased transcript levels of *Cyp6g1*, *Cyp6a2*, *hsp60*, *hsp70*, *grim*, *reaper*, and *Dmp53* genes (3.39, 1.85, 2.9, 1.71, 2.96, 5.49, and 1.95-fold higher than under control conditions, respectively), except for *hid*, which showed downregulated expression (0.19) (Figure 6). In contrast, ZEN administration to Oregon R(R)-flare larvae induced a different expression profile (Figure 6B). While *Cyp6g1* and *Cyp6a2* transcripts were induced by ZEN, the amount of *Cyp6a2* transcript was 16-fold higher than under the control condition. Interestingly, the oxidative stress genes showed a contrasting expression profile, ZEN increased the amount of *hsp60* transcript (1.69) but the expression level of *hsp70* decreased (0.5 times), compared to the control conditions. Finally, exposure to the mycotoxin increased the levels of *grim*, *hid*, and *Dmp53* (1.99, 1.21, and 1.82-fold, respectively), while the levels did not changer with *reaper*, compared to the control conditions (Figure 6B).

Thus, ZEN exposure modified the gene expression in *D. melanogaster* flare and Oregon R(R)-flare strains differently. The amounts of *Cyp6g1* and *Cyp6a2* transcripts were 4.5 and 3.0-fold higher in the Oregon R(R)-flare strain than in the flare strain when ZEN was added (Figure 6B). On the other hand, the relative expression levels of anti-apoptotic and pro-apoptotic *hsp60* and pro-apoptotic *hsp70* were similar (0.9 and 1.2, respectively) in both strains under the same conditions. Among the apoptosis and DNA damage markers the transcript levels of *hid* in the Oregon R(R)-flare strain were outstanding (19-fold higher than in the flare strain), while the expressions of the other genes were down-regulated (*grim* 0.4 and *reaper* 0.6) or similar (*Dmp53* 1.3) (Figure 6B).

### 2.8. Reactive Oxygen Species Quantification

**Figure 7:**
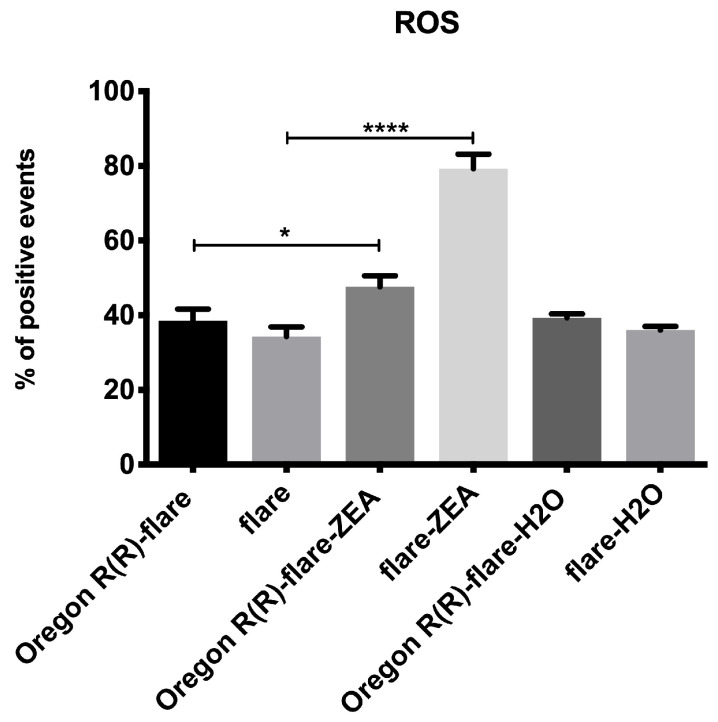
ROS levels induced by ZEN. Changes in the percentage of ROS-positive midgut cells in *D. melanogaster* flare and Oregon R(R)-flare larvae exposed to ZEN (260 μM) dissolved in PBS pH 7. The data are presented as the mean ± SD of three independent experiments. Asterisks denote statistical significance, with *p*-values of <0.005 * and <0.0001 ****, as determined by a two-tailed *t*-test (indicated by the capped line).

The redox state of larval midgut cells was directly measured by flow cytometry using 2′,7′-dichlorodihydrofluorescein diacetate (DCF-DA). ZEN exposure induced a statistically significant increase (*p* < 0.0001) in the percentage of positive events (Figure 7) in both strains. Interestingly, the increase in the Oregon R(R)-flare strain (in relation to the control) was less than that induced in the flare strain.

## 3. Discussion

ZEN’s toxicity was not demonstrated in the *D. melanogaster* flare and Oregon R(R)-flare strains through the survival assay, as mortality rates were not directly proportional to concentrations. Nonetheless, mortality did not exceed 30%. Therefore, we used a sublethal concentration (260 μM), which caused mortality between 20–25% in both strains, for the assays described, except for the *Drosophila* wing spot test. *D. melanogaster* possesses a steroid dehydrogenase [23] that can reduce ZEN to α-ZOL or β-ZOL metabolites, and the *Cyp1a2* and *Cyp6g1* genes, homologs to human *Cyp1A2* and *Cyp3A4* genes, respectively, that yield OH-ZEN [24]. Since the Oregon R(R)-flare strain showed high levels of *Cyp450*s [20] and a more efficient XM [25], a lower mortality (20% vs. 23%) was induced by ZEN (260 µM), in contrast with the flare strain, which had inducible *Cyp*450s levels that could account for the polynomial curves observed in the mortality rates at different concentrations (Figure 1).

The results of the *Drosophila* wing spot test in the ST and HB crosses suggested that ZEN and its metabolites did not have genotoxic effects, such as mutations, deletions, aneuploidy, or recombination. This contrasted with the findings of Abbès et al. [26], who observed an increase in chromosome aberrations in mouse bone-marrow cells exposed to ZEN and its metabolites (α-ZOL and β-ZOL) at higher concentrations than those used in our study. However, the ST cross showed a significant decrease in the number of, and altered distribution of, mwh clones at 100 and 400 µM treatments (Table 2). The mwh clones’ distribution results from cell division of imaginal disc cells of the third instar larvae, so the alteration of this process might have been due to a cytotoxic effect or apoptosis induction. All ZEN treatments, when compared to the dissolvent control (PBS), showed statistical differences, with a tendency for there to be as decrease in the mwh clones only in the ST cross. The statistical analyses of spot frequencies/fly and “mean mwh clone size class” were performed with the computer program SMART PC-version 2.1. To compare the “mean mwh clone size class” distributions of treated and control series statistically, we performed a one-sided Kolmogorov–Smirnov test (*p* level < 0.05). The “accumulated mwh clone size class” results showed significant differences between treated and both control series, indicating that cell division was disrupted by the parental compound or its metabolites. Therefore, these results indicated cytotoxicity, according to Santos-Cruz et al., and Graf et al. [21,22].

To confirm that *Drosophila* flare and Oregon R(R)-flare strains can incorporate ZEN, an HPLC analysis was conducted on adults that emerged from the treated larvae, which revealed that larvae from both strains incorporated similar amounts of ZEN. However, toxic effects, on development and reproduction, were observed, as evidenced by a decrease in imago emergence and a significant decrease in fecundity in both strains. The Oregon R(R)-flare strain exhibited higher sensitivity than the flare strain, with an emergence decrease of 27.7% and a 55% decrease in fecundity, compared to 20.8% and 39%, respectively, in the flare strain (Figure 2). This higher sensitivity may be related to Cyp450s enzyme levels, which could generate more OH-ZEN metabolites, as demonstrated by Bravin et al. using microsomes, and, thereby, exert effects on development [24].

It is worth noting that ZEN and its metabolites are known to bind to estrogen receptors [27,28], but since *D. melanogaster* lacks an estrogen receptor, the effects of ZEN on this route cannot be related to this receptor. However, as there are no known reported ligands for the ERR of *Drosophila*, it is possible that the chemical structure of OH–ZEN metabolites could bind to the orphan nuclear receptors, although this union and its effects on carbohydrate metabolism or testicular development must be demonstrated in the future [29,30].

It has been shown in human cell lines that ZEN modifies DNA global methylation patterns, as well as the expression profiles of some genes involved in cell metabolism [31]. In *Caenorhabditis elegans*, ZEN modifies the expression of a set of genes involved in collagen synthesis, which could account for disruption in its viability and reproduction [32]. This mycotoxin affected the viability of Sertoli cells in rats through arrest of primary cells and the triggering of autophagy [33]. On the other hand, ZEN metabolites have been proven to be involved in oxidative stress [34]. Heat shock proteins are key cellular responses to toxicant exposure and have been used as an indicator of short-term stress and toxicity [35]. We determined the relative expression levels of oxidative stress taking into account previous data for anti-apoptotic and pro-apoptotic *hsp60* and pro-apoptotic *hsp70*, apoptosis markers (*hid*, *grim* and *reaper*) and DNA damage (*Dmp53*) genes in the flare and Oregon R(R)-flare strains treated with ZEN [36,37,38]. Our results indicated that, even under control conditions, the genetic background of Oregon R(R)-flare included the overexpression of *hsp70* (4.9-fold higher than in the flare strain under similar conditions), *hid* (3.74), *reaper* (2.81) and *Dmp53* (1.37). The levels of *Cyp6g1* and *Cyp6a2*, which had been previously shown [25,39] were confirmed to be 10.3- and 3-fold higher, respectively (Figure 6).

Interestingly, our results demonstrated that ZEN treatment modified the expression profiles in both strains. In flare, the mycotoxin induced upregulation of all markers (*Cyp6g1*, *Cyp6a2*, *hsp60*, *hsp70*, *grim*, *reaper* and *Dmp53*) except *hid*, which was downregulated. The Oregon R(R)-flare strain expression profile was modified similarly, but, in this strain, *hsp70* was downregulated and *reaper* expression levels remained unchanged, compared to the dissolvent control condition. Proteins Hsp60 and Hsp70 are involved in the anti- and pro-apoptotic processes in *D. melanogaster*, specifically the Hsp60 family of chaperones, which have anti- and pro-apoptotic properties in the cytosol and organelles, predominantly in the mitochondria. In larvae, overexpression of this family of chaperones is located only in the Malpighian tubules and is induced by cell stress [40].

On the other hand, the Hsp70 stress-inducible protein mainly acts as an anti-apoptotic protein that inhibits apoptosis induced by cytotoxicity through chaperone-dependent, as well as independent, activities. However, the activity of the apoptotic caspase-activated DNase (CAD) is necessary [36]. Therefore, our ROS quantification results could provide evidence of oxidative cellular stress caused by ZEN metabolism, which, in turn, could induce apoptosis, especially in the flare strain, which lacks efficient detoxification Phase I and II XM, compared to the Oregon R(R)-flare strain [25,41]. We believe this assumption could be associated with the alteration of cell division in the imaginal disc cells, observed in the SMART wing test, as evidenced by the mwh clone size cytotoxic results. To further investigate the cellular processes involved in the metabolism induced by ZEN treatment, we analyzed the levels of some apoptosis biomarkers. In *Drosophila*, genes *reaper*, *grim* and *hid* (located in H99 locus) code for pro-apoptotic proteins, and their ectopic expression can induce apoptosis [42].

The relative expression levels of *grim* and *reaper* were increased in the flare strain by 2.96 and 5.49, respectively, indicating that ZEN, or its metabolites, induced the expression of these pro-apoptotic genes (Figure 6B). It has been reported that the transcription of *reaper*, *grim* and *hid* can be activated in response to many different signals, including steroid hormones, developmental signals, radiation, and various forms of cell stress or injury [43]. Therefore, our data support the proposal that cellular stress is induced by ZEN and/or its metabolites. Additionally, the increase in *Dmp53* expression levels and apoptosis transcripts in both strains was in agreement with the oxidative stress data presented, indicating the induction of imbalance in oxidative radicals and the triggering of DNA repair or cell cycle death by ZEN and/or its metabolites =. Therefore, we propose that the toxicity of ZEN in this study was due to its oxidative damage, since *Drosophila* does not have an estrogen receptor [11].

In summary, the results of our study indicate that ZEN does not exhibit genotoxic activity in the *Drosophila* wing spot test, but it does affect cell division, as evidenced by the distribution of mwh clones in the flare strain, as well as emergency and fecundity in both strains. This effect cannot be attributed to ZEN or its metabolites interacting with the estrogen receptor, as *Drosophila* lacks a homologous receptor. Instead, other mechanisms not yet reported must be involved. Our study also showed that ZEN exposure increased the expression levels of anti-apoptotic and pro-apoptotic *hsp60* in both strains and modulated the cell pathways of apoptosis gene transcripts (*reaper* in the flare strain and *grim* in both strains), suggesting an increase in ROS and supporting cytotoxic activity. Additionally, we demonstrated that ZEN exposure induced the expression of *Cyp6a2* and *Cyp6g1*, indicating that these enzymes are involved in ZEN metabolism in this model organism and may explain the cytotoxicity, oxidative imbalance, and development and fecundity alterations observed in our study.

## 4. Materials and Methods

### 4.1. T Chemicals and Regents

Zearalenone (ZEN, CAS-No. 17924-92-4), sodium phosphate monobasic monohydrate (CAS-No. 10049-21-5), urethane (URE, CAS-No. 51-79-6), toluene (CAS-No. 108-88-3), and ethidium bromide (CAS-No. 214-984-6) were purchased from Sigma Aldrich (St. Louis, MO, USA). Ethanol (EtOH, CAS-No. 64-17-5) was purchased from Merck and Darmstadt Co. *Drosophila* Instant Medium (DIM), Formula 4-24 was purchased from North Carolina Biological Supply Co., (Whitsett, NC, USA). Platinum DNA polymerase (Cat. No. 11304011), Oligo (dT)20 Primer (Cat. No. 12577011), and TRIzol reagent (CAT No. 15596-018) were purchased from Invitrogen Co. (Carlsbad, CA, USA). The Revert Aid First Strand cDNA Synthesis kit (San Diego, CA, USACAT K 1622) and MAXIMA SYBR Green/ROX Master Mix (2X) (San Diego, CA, USA CAT K0222) were acquired from Thermo Fisher Scientific.

### 4.2. Drosophila Strains

*Drosophila melanogaster* mutant strains flare (flr3/In(3LR)TM3,ri pp sepl(3)89Aa bx34e e BdSer), Oregon R(R)-flare (ORR(1);ORR(2);flr/In(3LR)TM3,ri pp sepl(3)89Aa bx34e e BdS), and multiple wing hairs (mwh/mwh), originally donated by Dr. Ulrich Graf (ETH, Zurich), were used in this study. The phenotypic markers of the strains were verified continuously [44]. The flies were cultured in media containing 5 g of potato flakes (Maggi^®^, Nestlé, Glendale, CA, USA) and 20 mL of a preservative solution [99 mL of tap water added to 0.5 mL of a 12% ethanol solution of methyl 4-hydroxybenzoate and 0.5 mL of a 10:1 solution of propionic acid: orthophosphoric acid] [45].

### 4.3. Survival Assays (LC_50_)

A Lethal media concentration assay (LC_50_) assay was conducted for ZEN in *Drosophila melanogaster* flare and Oregon R(R)-flare strains, following the protocol described by Castañeda et al. [46]. The flies were reared in 250 mL flasks with a culture medium at 25 °C and 65% relative humidity [45]. Eggs were collected for 8 h at 25 °C, 65% RH, and in the dark, in flasks containing fermenting fresh baker’s yeast supplemented with sucrose and water. The eggs were then incubated under the same conditions. Three days later, the third instar larvae (72 ± 4 h) were washed out of the bottles with tap water (25 °C) through a fine-meshed stainless-steel strainer. Subsequently, 10 larvae were transferred to vials containing 0.5 g of *Drosophila* Instant Medium (DIM), supplemented with 2 mL of ZEN solutions [100, 200, 300, and 500 μM] in potassium phosphate monobasic buffer 150 mM, pH 7 (PBS). The treatment vials were kept at 25 °C, 65% RH, and were conducted in the dark, to complete their development. The surviving adult flies were counted and collected from the vials 10 to 12 days after the eggs were collected. The LC50 data obtained from four independent experiments (with three replicates per treatment) were analyzed using a two-way ANOVA at a significance level of *p* < 0.05 (SPSS software vs. 2, IBM, New York, NY, USA) (Supplementary Information). Since a 100% mortality rate was not reached, ZEN was also tested at 260 μM, a sublethal concentration that corresponded to a 20–25% mortality rate in both strains.

### 4.4. Drosophila Wing Spot Test

Virgin females from the flare and Oregon R(R)-flare strains were mated with males from the multiple wing hair strain to perform standard (ST) and high bioactivation (HB) crosses, respectively [47]. Eggs were collected using the method described earlier, and third instar larvae (72 ± 4 h) were transferred to vials containing 0.5 g of DIM added with 2.0 mL of negative control (milliQ water), dissolvent control (PBS), pro-mutagen bioactivation positive control (urethane, 20 mM), or ZEN dissolved in PBS (100, 200 and 400 μM) as treatments. The vials were cultured under dark conditions at 25 °C and 65% relative humidity until imago emergence. Surviving flies were collected and stored in 70% EtOH.

To assess the mutation frequency, wild type wings (trans-heterozygous, mwh +/+ flr3) of flies from the two crosses were mounted on microscope slides using Entellan^®^ solution, and scored for unbiased observation under a microscope at 400× magnification, where areas with morphological mutations were visible as clone spots. The frequency of each type of spot (small and large single spots or twin spots) and the total spot frequency per treatment [21] were compared pairwise using unpublished SMART computer software [48], based on the Kastenbaum–Bowman test (*p* < 0.05) [49] and the Mann–Whitney U test, using the STAT Graphics version 6.0 software (Statgraphics Technologies, The Plains, Virginia, USA) (*p* < 0.05).

Furthermore, the Kolmogorov–Smirnov test was performed to statistically analyze the accumulated mwh clone size distribution in each treatment against the corresponding control (*p* < 0.05). This test indicated significant alteration of the mitotic division on imaginal wing cells, and, therefore, indicated cytotoxic effects [21,22].

### 4.5. High Performance Liquid Cromatography (HPLC) Analysis

An HPLC analysis was performed in three independent assays to detect and quantify the remaining ZEN in imagos obtained from flare and Oregon R(R)-flare strains that were treated with ZEN. For this purpose, third instar larvae (72 ± 4 h) from each strain were fed with medium supplemented with ZEN (260 µM), or not so supplemented, for approximately 48 h, and 15 imagos were ground in methanol (500 µL). The samples were centrifuged to remove cell debris, and the supernatant was collected for analysis. The HPLC was carried out in accordance with the method described by Visconti and Pascale [50], with an Agilent 1100 system (Hewlett Packard) equipped with the following: a manual injector (Agilent Technologies) with a 20 μL loop, a degasser (Agilent), a pump (Agilent), and a fluorescence detector LS50B (Perkin Elmer). The chromatographic conditions were as follows: the mobile phase consisted of a mixture of methanol: acetic acid 1% 62:38 (*v/v*), the flow rate was 1 mL/min, the Supelcosil LC-18 column (250 mm × 4.6 mm) was kept at room temperature, and the fluorescence detector was set at excitation and emission wavelengths of 280 and 460 nm, respectively. The detection limit of the method was 8.0902 ng and the limit of quantification was 22.608 ng.

### 4.6. Emergence

Third instar larvae (72 ± 4 h) from the flare and Oregon R(R)-flare strains were transferred separately to vials containing 0.5 g of DIM with 2 mL of PBS as a dissolvent control (10 organisms per vial and five vials per treatment). Another group of organisms was placed in vials containing 0.5 g of DIM with 2 mL of toluene (TOL, 50 mM) dissolved in PBS, serving as a positive control [51]. Finally, a third group of larvae was placed in vials with 0.5 g of DIM added to 2.0 mL of ZEN (260 μM) dissolved in PBS. All larvae were maintained in their respective treatments for ~48 h and then transferred to vials containing 0.5 g of DIM added with 2 mL of milliQ water. These vials were monitored daily, and the imagos were collected and counted until all emerged. The total number of imagos was considered to be the corresponding emergence for each treatment. The data obtained from these experiments were analyzed with a two-way ANOVA at a significance level of *p* < 0.05, using the SPSS software version 2 (IBM, New York, NY, USA).

### 4.7. Fecundity

Fecundity was assessed by maintaining the surviving imagos from each strain and treating the previous emergence assay in vials containing 0.5 g DIM added with 2.0 mL milliQ water. Virgin female flies were selected, and ten individual pairs of females and males were placed in empty vials for 5–6 h. Each pair was then transferred to a modified vial with an upside-down tap, filled with pulverized DIM 0.5 g added to 2.0 mL milliQ water and food colorant. The number of eggs laid by each female was recorded daily for ten days, and the percentage of eggs laid per female was considered to be fecundity. The data obtained from these experiments were analyzed using a two-way ANOVA with a significance level of *p* < 0.05 (SPSS software version 2).

### 4.8. Total RNA Isolation and cDNA Synthesis

Batches of third instar larvae (72 ± 4 h) from the flare and Oregon R(R)-flare strains were fed for approximately 48 h with a mixture of 0.5 g of DIM and 2.0 mL of ZEN 260 μM dissolved in PBS. The larvae were immediately immersed in liquid nitrogen and transferred to an ultra-low freezer at −70 °C for future molecular assays. Total RNA was extracted using TRIzol reagent, chloroform, isopropanol, and ethanol, following the manufacturer’s instructions (Invitrogen, Carlsbad, CA, USA). The extracted RNA was diluted in 50 μL of RNase-free water and treated with DNase (Turbo DNA-free, Invitrogen) to ensure its integrity. The RNA was quantified in a spectrophotometer (Nano Drop 2000 UV-Vis, Thermo Scientific, San Diego, CA, USA) and stored at −70 °C for future use. The cDNA was synthesized from 2 μg of total RNA using reverse transcriptase reaction with oligo (dT)20 primer and a Revert Aid First Strand cDNA Synthesis kit from Thermo Scientific, following the manufacturer’s instructions. The reaction mixture was incubated at 42 °C for 90 min.

### 4.9. Real Time Semi-Quantitative PCR

Specific primers for all genes were designed using primer3-Blast software and are listed in Table 3. PCR reactions were performed in triplicate using 0.5 mM of the specific primers, Maxima SYBRGreen/Rox 10 qPCR Master Mix (Thermo Scientific), and 1 μL of cDNA. The amplification reaction was carried out in a Step One thermocycler (Applied Biosystems) with the following thermal profile: 95 °C/10 min followed by 40 cycles of 95 °C/30 s and 60 °C/60 s. The relative expressions of *Cyp6g1*, *Cyp6a2*, *hsp60*, *hsp70*, *grim*, *hid*, *reaper*, and *Dmp53* were evaluated using the 2^−∆∆Ct^ method, with *b-actin* serving as the housekeeping gene. For basal (untreated) expressions, the flare strain values (Figure 6A) were used as the reference sample in the 2^−∆∆Ct^ analysis. For the expression analysis in the presence of ZEA, the corresponding sample (either flare or Oregon R(R)-flare, as appropriate) treated with the control (PBS pH 7) served as the reference sample.

### 4.10. Reactive Oxygen Species Quantification

Third instar larvae (72 ± 4 h) from both strains were collected and transferred to flasks containing 3 g of DIM, supplemented with 12 mL of ZEN (260 μM) dissolved in PBS. PBS was used as a dissolvent and negative control, while Milli Q water was used as a negative control. The treatment flasks were maintained at 25 °C, with a relative humidity of 65% and a 12:12 h light–dark cycle for 24 h. After this, the larvae (96 ± 4 h) from each treatment were washed thoroughly with tap water at room temperature. Batches of larvae from both strains per treatment were used for flow cytometry analysis of ROS in midgut cells.

For the preparation of tissue homogenate, midguts were collected from 20 larvae of each strain and incubated in collagenase (0.5 mg/mL) (Sigma-Aldrich, St. Louis, MO, USA) for 15 min at 24 ± 1 °C. The cells were then passed through a nylon mesh (85 μm), washed with phosphate-buffered saline (PBS, pH 7.4, 4 °C) to remove collagenase, and processed for the measurement of ROS generation. This protocol was adapted from a previously described method [52].

The intracellular ROS levels in midgut cells of each strain were determined using the fluorescent probe 2′,7′-dichlorodihydrofluorescein diacetate (DCF-DA) (Sigma Chemicals, St. Louis, MO, USA) and flow cytometry, following the protocol described by Eruslanov and Kusmartsev [53]. The obtained data were analyzed using FlowJo software v10.9 (Veritas), and 30,000 events were counted per sample.

## Figures and Tables

**Table 1:** Summary of the wing spot test. The results for MilliQ water (W), PBS buffer (PBS), urethane (URE), and zearalenone (ZEN) in the standard (ST) and High Bioactivation (HB) crosses are presented after scoring marker-heterozygous flies (mwh+/+flr3 or Oregon R(R); mwh+/+flr3) wild-type wings.

Compound Cross ^b^ Type	Conc. (M, mM)	Number of Flies	Spots per Fly (Number of Spots) Statistical Diagnosis ^a^													Mean mwh Clone Size Class	Clone Formation Per 10^5^ Cells Per Cell Division ^c^	
			Small Single Spots (1–2 cells) m = 2			Large Single Spots (>2 cells) m = 5			Twin Spots m = 5			Total Spots m = 2			mwh Clones		Observed	Control Corrected
Negative, dissolvent and positive controls																		
ST																		
WATER	0	61	0.74	(045)		0.07	(004)		0.07	(004)		0.87	(053)		52	1.52	1.75	
PBS	0	59	0.95	(056)	−	0.15	(009)	−	0.07	(004)	−	1.17	(069)	−	68	1.71	2.35	0.60
URE	20	38	3.13	(119)	+	0.24	(009)	+	0.05	(002)	−	3.42	(130)	+	129	1.42	6.95	5.20
HB																		
WATER	0	47	0.77	(036)		0.09	(004)		0.02	(001)		0.87	(041)		39	1.33	1.70	
PBS	0	55	0.65	(036)	−	0.13	(007)	−	0.00	(000)	−	0.78	(043)	−	42	1.55	1.55	−0.15
URE	20	39	6.31	(246)	+	0.62	(024)	+	0.26	(010)	+	7.18	(280)	+	277	1.59	14.55	12.85
Treatments ZEN																		
ST																		
PBS	0	59	0.95	(056)		0.15	(009)		0.07	(004)		1.17	(069)		68	1.71	2.35	
ZEN 1	100	55	1.15	(063)	−	0.05	(003)	−	0.09	(005)	−	1.29	(071)	−	71	1.44	2.65	0.30
ZEN 2	200	58	0.95	(053)	−	0.14	(008)	−	0.03	(002)	−	1.09	(063)	−	63	1.81	2.20	−0.15
ZEN 3	400	55	0.73	(040)	−	0.20	(011)	−	0.00	(000)	−	0.93	(051)	−	49	1.76	1.80	−0.55
HB																		
PBS	0	55	0.65	(036)		0.13	(007)		0.00	(000)		0.78	(043)		42	1.55	1.55	
ZEN 1	100	55	0.75	(041)	−	0.09	(005)	−	0.00	(000)	−	0.84	(046)	−	39	1.67	1.45	−0.10
ZEN 2	200	56	0.73	(041)	−	0.11	(006)	−	0.02	(001)	−	0.86	(048)	−	48	1.85	1.75	0.20
ZEN 3	400	56	0.86	(048)	−	0.09	(005)	−	0.00	(000)	−	0.95	(053)	−	50	1.62	1.80	0.25

^a^ Statistical diagnosis. m: minimal risk multiplication factor for the assessment of negative results. For the final statistical diagnosis of all positive (+) and negative (−) outcomes, the non-parametric Mann–Whitney and Wilcoxon U-test, with significance α levels and β, were used to exclude false positive or negative diagnoses. One side binomial test with the following α and β significative results: + (α ≤ 0.05; no significative results: − (β ≤ 0.05). ^b^ ST: standard cross; HB: high bioactivation cross. ^c^ Clone frequencies per fly were divided by the number of cells examined per fly (48,800) to give an estimate of formation frequencies per cell and per cell division in chronic exposure experiments.

**Table 2:** Results of the wing spot test. The test was performed on milliQ water (W), phosphate monobasic buffer pH 7 (PBS), urethane (URE 20 mM), and zearalenone (ZEN) in the standard (ST) and High Bioactivation (HB) crosses of the Drosophila wing spot test, and the results were analyzed by accumulated mwh clone size class distribution analyses using the Kolmogorov–Smirnov test. SD represents the standard deviation, and a *p* value of less than 0.05 was considered statistically significant.

	ST Cross			HB Cross		
	Mean	SD	Mean	SD	Mean	SD
Controls						
WATER	51.25	4.80		48.12	5.33	
PBS	59.75	8.89	<0.025	39.75	5.34	<0.005
URE (20 mM)	121.87	12.84	0.000	252.00	36.88	0.000
Treatments ZEN (mM)						
PBS	59.75	8.89		39.75	5.34	
ZEN 100	63.62	5.60	<0.025	42.25	6.34	<0.025
ZEN 200	55.37	8.78	<0.025	42.25	7.63	<0.025
ZEN 400	46.00	6.93	<0.005	49.00	7.42	<0.005

**Table 3:** Sequences of Oligonucleotides. This table presents the sequences for both forward and reverse oligonucleotides, along with their corresponding alignment temperatures and amplicon lengths.

Gene	Oligonucleotide Sequence (Forward and Reverse)	Annealing Temperature (°C)	Amplicon Length (bp)
*Cyp6g1*	GAGCCTGAAGCCGTTCTAC ATCCGAAGGGTTGATATGCC	60	176
*Cyp6a2*	CGGAAAGAAGTGGAAGGAC CACATCGGTGGTGAACCTG	60	194
*hsp60*	GAGACCGTCAAGGACAACC CCTCGCTGATGAGATTGCC	60	121
*hsp70*	CGAGATTGACGCACTGTTTG GCCGACGAGACGATGTC	60	168
*grim*	GGGAAGTCAACAGGGATCG CCTTGGAGGTGGCATCGG	60	197
*hid*	GAGTGGGTCAGGATGTACC GAGTTCGGATTCGGATGGC	60	166
*reaper*	CTACATACCCGATCAGGCG CGATGGCTTGCGATATTTGC	60	177
*mp53*	GCACTTCAGCCAGCAATCC CCACCGATGTTGTGATTCTC	60	110
*actin*	GTCCCTGGAGAAGTCGTAC GCACAGTGTTGGCGTACAG	60	194

## Data Availability

The data is unavailable due to privacy restrictions.

## Supplementary Materials

The following supporting information can be downloaded at: https://www.mdpi.com/article/10.3390/toxins15060358/s1, Figure S1: ZEN detection and quantification. (A) HPLC Chromatogram showing ZEN in D. melanogaster larvae and (B) quantification of ZEN in larvae of D. melanogaster flare and Oregon R(R)-flare strains. Third instar larvae (72 ± 4 h) from each strain were fed with medium supplemented with ZEN 260 μM for ~48 h and 15 imagos were groud in methanol and analyzed by HPLC (see methods).; Table S1: Imago mortality for both strains after feeding larvae with different concentrations of ZEN. Data obtained from four independent experiments with three replicates per treatments (see Methods).
