# Supplementary material for: Zearalenone Does Not Show Genotoxic Effects in the Drosophila melanogaster Wing Spot Test, but It Induces Oxidative Imbalance, Development, and Fecundity Alterations
Source: Toxins (Basel). 2023 May 25;15(6):358. doi: 10.3390/toxins15060358 (PMC10301386; doi:10.3390/toxins15060358)
Supplement: Supplementary file 1 [file toxins-15-00358-s001.zip › toxins-2408362-supplementary.pdf]

toxins

MDPI

A B

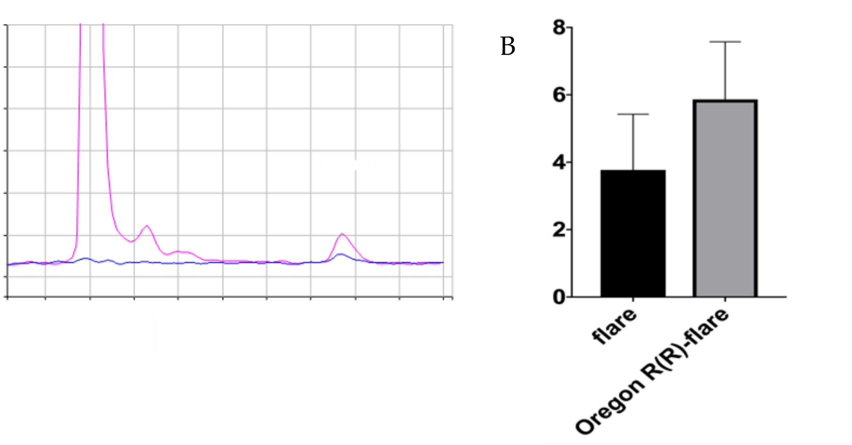

Figure S1. ZEN detection and quantification. (**A**) HPLC Chromatogram showing ZEN in D. melanogaster larvae and (**B**) quantification of ZEN in larvae of D. melanogaster flare and Oregon R(R)-flare strains. Third instar larvae (72 ± 4 h) from each strain were fed with medium supplemented with ZEN 260 μM for ~48 h and 15 imagos were grinded in methanol and analyzed by HPLC (see methods).

Table S1. Imagos mortality for both strains after feeding larvae with different concentrations of ZEN. Data obtained from four independent experiments with three replicates per treatments (see Methods).

	Drosophila melanogaster			
	**flare**		**Oregon R(R)-flare**	
ZEN	Mean	SD	Mean	SD
0	6.67	4.71	5	1.92
100	8.33	4.30	8.33	4.30
200	27.50	1.67	12.50	1.67
300	21.67	6.38	26.67	7.20
500	12.50	9.95	20.00	9.81
